# Chronic Ketamine Reduces the Peak Frequency of Gamma Oscillations in Mouse Prefrontal Cortex *Ex vivo*

**DOI:** 10.3389/fpsyt.2013.00106

**Published:** 2013-09-17

**Authors:** James M. McNally, Robert W. McCarley, Ritchie E. Brown

**Affiliations:** ^1^Laboratory of Neuroscience, Department of Psychiatry, VA Boston Healthcare System, Harvard Medical School, Brockton, MA, USA

**Keywords:** gamma oscillations, NMDA receptors, ketamine, schizophrenia, prefrontal cortex

## Abstract

Abnormalities in EEG gamma band oscillations (GBO, 30–80 Hz) serve as a prominent biomarker of schizophrenia (Sz), associated with positive, negative, and cognitive symptoms. Chronic, subanesthetic administration of antagonists of *N*-methyl-D-aspartate receptors (NMDAR), such as ketamine, elicits behavioral effects, and alterations in cortical interneurons similar to those observed in Sz. However, the chronic effects of ketamine on neocortical GBO are unknown. Thus, here we examine the effects of chronic (five daily i.p. injections) application of ketamine (5 and 30 mg/kg) and the more specific NMDAR antagonist, MK-801 (0.02, 0.5, and 2 mg/kg), on neocortical GBO *ex vivo*. Oscillations were generated by focal application of the glutamate receptor agonist, kainate (KA), in coronal brain slices containing the prelimbic cortex. This region constitutes the rodent analog of the human dorsolateral prefrontal cortex, a brain region strongly implicated in Sz-pathophysiology. Here we report the novel finding that chronic ketamine elicits a reduction in the peak oscillatory frequency of KA-elicited oscillations (from 47 to 40 Hz at 30 mg/kg). Moreover, the power of GBO in the 40–50 Hz band was reduced. These findings are reminiscent of both the reduced resonance frequency and power of cortical oscillations observed in Sz clinical studies. Surprisingly, MK-801 had no significant effect, suggesting care is needed when equating Sz-like behavioral effects elicited by different NMDAR antagonists to alterations in GBO activity. We conclude that chronic ketamine in the mouse mimics GBO abnormalities observed in Sz patients. Use of this *ex vivo* slice model may be useful in testing therapeutic compounds which rescue these GBO abnormalities.

## Introduction

Mounting evidence suggests that the symptoms of neuropsychiatric disorders, such as schizophrenia (Sz), arise from a failure of the brain to properly integrate activity across local and distributed neuronal circuitry (1–3). Neuronal oscillations represent an essential mechanism responsible for such neural integration, providing temporal coordination of neuronal activity (4). In Sz patients, numerous clinical studies have observed abnormalities in oscillatory processes (5), particularly those in the gamma frequency band (gamma band oscillations, GBO; 30–80 Hz). GBO activity has been suggested to be critical for a number of sensory and cognitive tasks (6). As such, impaired GBO, particularly in prefrontal cortical regions, likely underlie positive, negative, and cognitive symptoms in Sz (3, 7).

Over the last few decades, administration of psychotomimetic agents, such as the *N*-methyl-d-aspartate receptor (NMDAR) antagonist, ketamine, have provided the most reliable and widely used means to mimic Sz-like symptoms, and currently represents the “gold-standard” for modeling this disorder in both humans, and animals (8–10). Recent *in vivo* rodent studies have shown that *acute* systemic administration of NMDAR antagonists leads to a significant potentiation of spontaneous GBO activity in frontal cortex (11–13). Such findings have been largely confirmed *ex vivo* by our lab and others (14, 15). However, *chronic* application of NMDAR antagonists arguably represents a more useful means to model Sz, since chronic administration causes structural alterations in neocortical circuitry similar to those observed in Sz patients (16, 17). However, to what extent chronic administration of ketamine and other NMDA receptor antagonists mimic Sz-like GBO abnormalities is not well understood. Thus, here we explore the effect of systemic, chronic administration of ketamine and the more specific NMDAR antagonist, MK-801 on GBO. In order to focus on changes occurring specifically in the neocortical circuitry, experiments were performed *ex vivo*, in slices containing the mouse prelimbic cortex (PrL), the rodent analog of the human dorsolateral prefrontal cortex (18), a region heavily implicated in many of the cognitive impairments associated with Sz (19, 20). GBO were elicited in submerged neocortical slices using our established method (15) utilizing brief, focal application of the glutamate receptor agonist kainate (KA).

## Materials and Methods

### Animals

Adult (>P91) heterozygous GAD67-GFP “knock-in” mice (Swiss Webster background), which express GFP under control of the promoter for GAD67 (21) of either sex were utilized for this work. As determined previously in McNally et al. (15), there are no significant sex-dependent differences in KA-elicited GBO in these mice. While we did not take specific advantage of the GFP labeling in the GAD67-GFP line in this study, our previous work examining the effects of acute NMDAR antagonist treatment on GBO activity was performed using this mouse line. Thus, we utilized the same line for the chronic studies reported here, allowing us to more accurately compare our present findings to those reported earlier. Mice were housed at the VA Boston Healthcare System, Brockton campus under constant temperature (23°C) and a 12 h:12 h light–dark cycle with food and water available *ad libitum*. All experiments were carried out in accordance with the American Association for Accreditation of Laboratory Animal Care’s policy on care and use of laboratory animals and were approved by the local Institutional Animal Care and Use Committee.

Chronic drug administration was conducted similar to the manner described in Behrens et al. (22). Mice were given daily i.p. injections of either ketamine-HCl (5 or 30 mg/kg), MK-801 (0.02, 0.5 or 2 mg/kg), or an equivalent volume of saline (<0.5 mL) for 5 days. The efficacy of these dosages for producing altered interneuronal parvalbumin and/or GAD67 expression reminiscent of that seen in postmortem Sz brains was determined in previous studies (16, 23–25). Mice injected with NMDAR antagonists exhibited increased locomotion for 1–2 h following treatment, as would be expected given the previous behavioral literature using these agents (8). However, this behavioral response was not characterized in detail. Twenty-four hours following the final drug/saline injection, animals were sacrificed and utilized as described below. Injectable Ketamine-HCl was obtained from Bioniche Pharma (Galway, Ireland), and MK-801 from Ascent Scientific (Bristol, UK).

### Slice preparation

Coronal slices containing the PrL were prepared as previously described in McNally et al. (15). Briefly, mice were deeply anesthetized using isoflurane, then quickly decapitated. The brain was removed and placed into ice cold modified artificial cerebrospinal fluid (ACSF) containing: (in millimoles) 252 Sucrose, 1.8 KCl, 1.2 KH_2_PO_4_, 2 MgSO_4_, 25.6 NaHCO_3_, and 10 glucose saturated with 95% O_2_/5% CO_2_. 450 μm slices were cut between +2.96 and +1.54 mm with respect to bregma [according to the Franklin/Paxinos atlas (26)] using a Vibratome 3000 (Vibratome, Bannockburn, IL, USA). Slices were then transferred into a prechamber (BSC-PC; Warner Instruments) containing ACSF: (in millimoles) 124 NaCl, 1.8 KCl, 1.2 KH_2_PO_4_, 2 CaCl_2_, 1.3 MgSO_4_, 25.6 NaHCO_3_, and 10 glucose, continuously bubbled with 95% O_2_/5% CO_2_ (pH 7.4). Slices were allowed to recover for at least 1 h before use. For recording, slices were transferred to a submersion-style recording chamber (RC27L; Warner Instruments) and constantly perfused (5 mL/min) with warm ACSF (30°C).

### Elicitation of GBO *in vitro*

As described in McNally et al. (15), extracellular field potential activity was recorded using glass micropipettes (2–5 MΩ) filled with ACSF and positioned ∼50 μm deep in the PrL (Layer II/III). Oscillatory activity was elicited by a brief (80 ms @ 30 psi) focal application of KA (1 mM) onto the PrL slice in close apposition to the location of the field potential electrode using a picospritzer (General Valve Corp.). Field potentials elicited by KA application were amplified using the 100× gain DC-coupled current-clamp mode of a Multiclamp700B amplifier (Axon Instruments). Signals were digitized at 10 kHz using a Digidata 1322A 16-bit data acquisition system (Axon Instruments), then filtered between 1 kHz and 0.1 Hz using pClamp 9.2 (Axon Instruments) and stored on a PC hard drive.

### Analysis of KA-elicited oscillations

**Figure 1:**
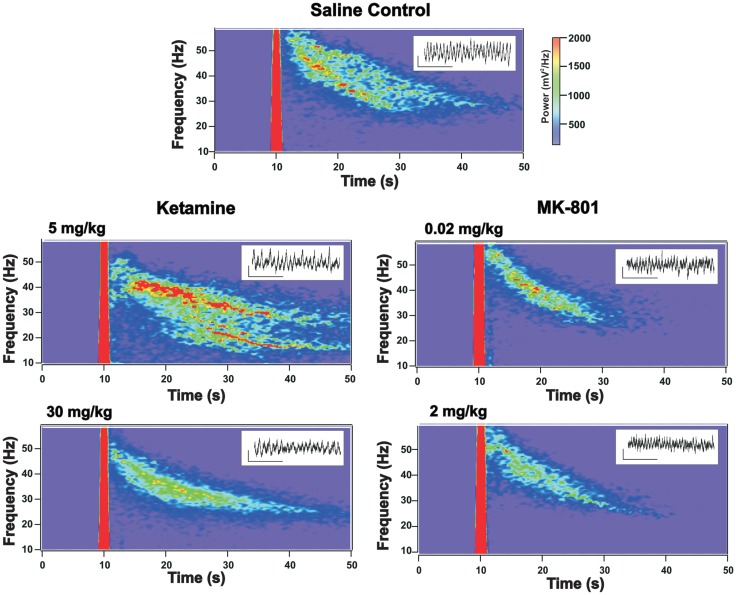
**Grand average of kainate (KA) elicited oscillatory response in prelimbic cortex (PrL) slices prepared from mice receiving chronic administration of NMDAR antagonists**. Time-frequency spectrograms show the average KA-elicited oscillatory response from acute PrL slices obtained from mice receiving chronic injections of saline (control), ketamine (5 and 30 mg/kg), or MK-801 (0.02 and 2 mg/kg). Note: the mechanical transient associated with KA application appears as a thick red line due to oversaturation. Compared to saline treated controls, the elicited response in slices from mice chronically treated with 30 mg/kg ketamine shows a reduction in the peak frequency (see Figure 2) and in the power in the (40–50 Hz) band, which is almost absent in slices from drug treated mice 5–10 s following KA application. 5 mg/kg ketamine treatment also appears to show a slight decrease in the higher-frequency elicited response, while the response at lower frequency bands appears elevated (not significant). MK-801 treated mice show a trend toward reduced power but no change in peak frequency (see Figure 2 and text). Insets provide representative examples of GBO signal traces recorded from acute slices from mice in each treatment group (scale bar for insets: *x* = 200 ms, *y* = 50 μV).

**Figure 2:**
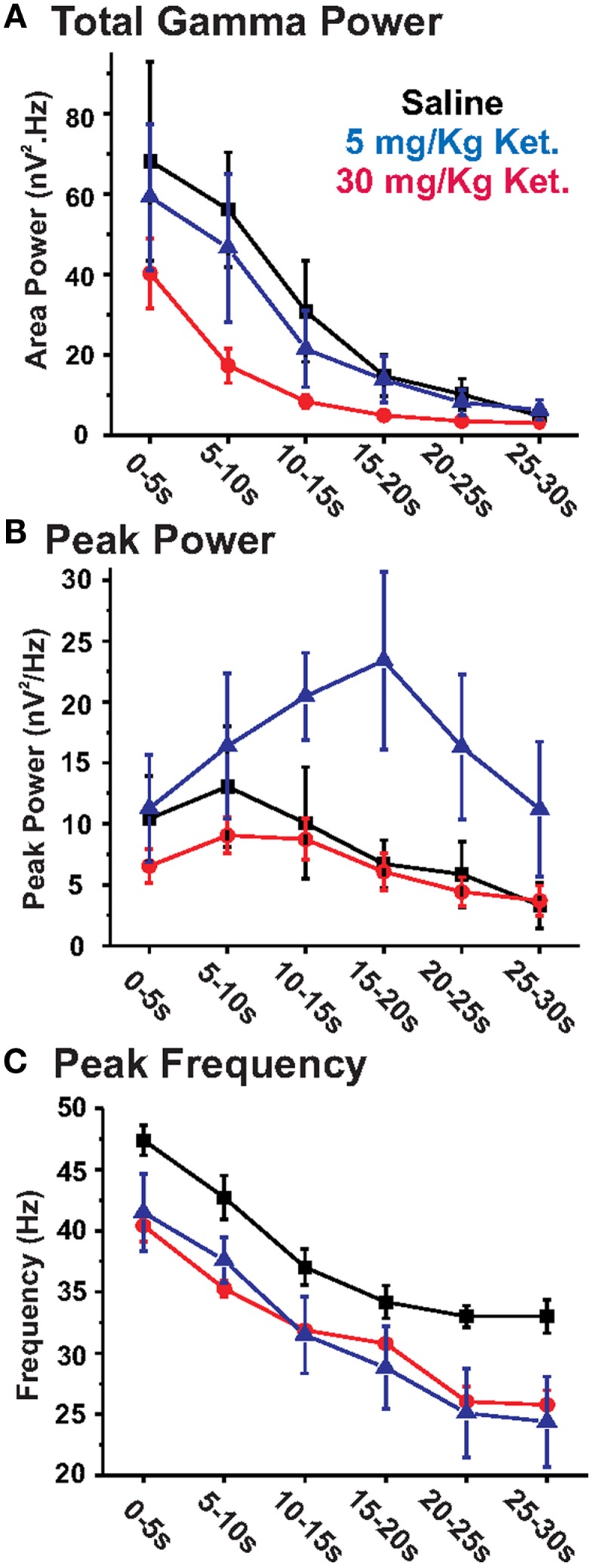
**Chronic ketamine treatment leads to decreases in the power and peak frequency of KA-elicited PrL oscillations**. Graphs show the effects of chronic ketamine given at daily doses of 5 mg/kg (blue) or 30 mg/kg (red) on **(A)** total GBO power (40–50 Hz), **(B)** peak power, and **(C)** peak frequency across 30 s of the elicited oscillatory response recorded from acute PrL slices. Compared to saline treated controls (black), mice receiving 30 mg/kg ketamine showed a significant decrease in both total GBO power (between 5 and 20 s; see Table 1) and the peak frequency of elicited activity. No change was observed in peak power. At 5 mg/kg, chronic ketamine had no significant effect on total GBO power or peak power. While a decrease in peak frequency was still apparent at this concentration, this effect was only significant in the 20–25 and 25–30 s epochs.

**Figure 3:**
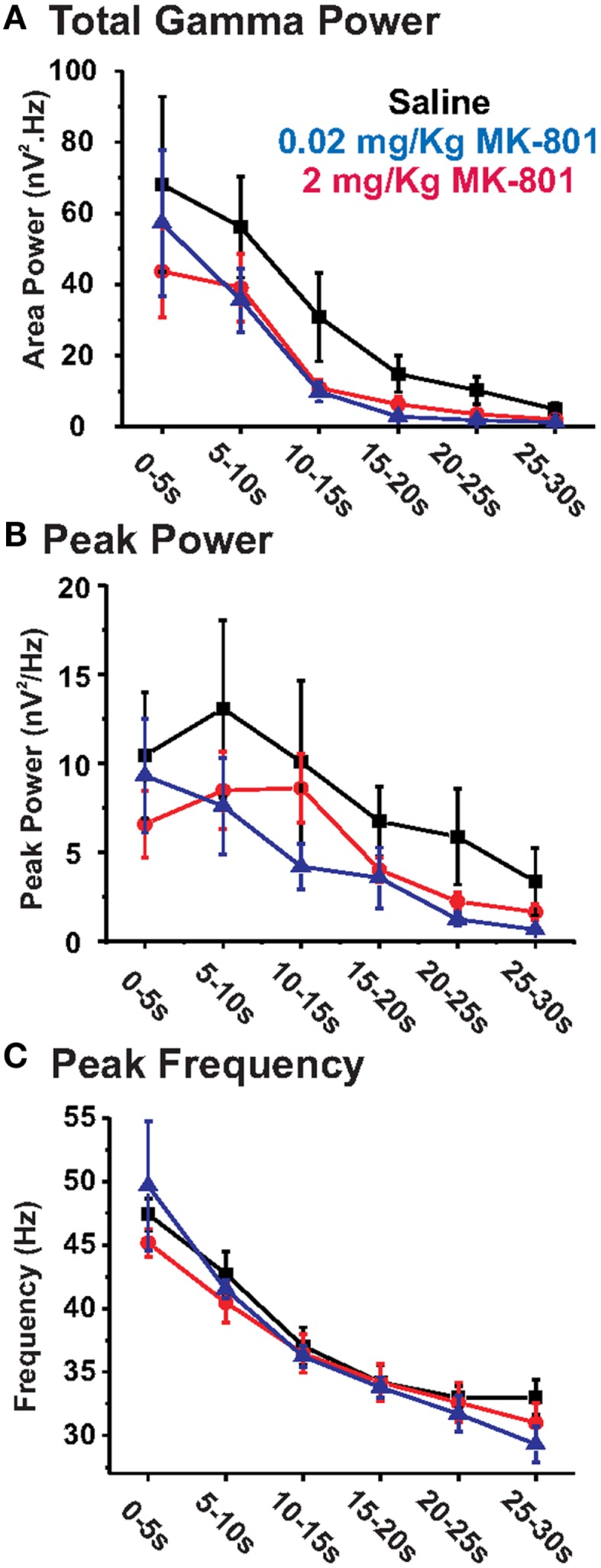
**Chronic MK-801 treatment does not significantly alter KA-elicited PrL oscillations**. Graphs show the effects of chronic MK-801 given at daily doses of 0.02 mg/kg (blue) or 2 mg/kg (red) on **(A)** total GBO power (40–50 Hz), **(B)** peak power, and **(C)** peak frequency across 30 s of the elicited oscillatory response recorded from acute PrL slices, compared to saline treated controls (black). At both concentrations chronic MK-801 treatment resulted in no significant alteration of KA-elicited oscillatory activity.

Kainate-elicited oscillations were characterized using both time-frequency, and power spectral density (PSD) analysis. Grand average time-frequency plots (Figure 1) were generated in Igor Pro (Wavemetrics), by performing short-time Fourier transform analysis (1 Hz resolution) on individual LFP records of elicited oscillatory activity. Time-frequency data was then averaged across all animals in each treatment group. For data presented in Figures 2 and 3, PSD profiles were generated by Fourier transform analysis of field potential recordings using both Clampfit (axon) and Igor Pro. PSD were calculated from a 30 s epoch of the field potential trace starting 2.5 s following application of KA, following dissipation of the mechanically evoked transient. KA-elicited oscillations were generated three consecutive times at 5 min intervals. Only slices yielding consistent PSD profiles across all trials (<10% difference in peak power, frequency) were used for analysis. To analyze the time course of oscillatory activity, the 30 s epoch of KA-elicited activity was broken up into six 5 s epochs; PSD profiles (1.2 Hz resolution) for each epoch were generated, and then averaged across the three trials to provide the average PSD over each epoch for each slice. Recordings were performed on one to three slices from each experimental animal. For each animal, PSD data from individual slices were averaged to give the average PSD profile for each animal. These values were then used for comparison between treatment groups.

**Table 1:** **Chronic NMDAR antagonist effects on evoked PrL GBO activity**.

Epoch	Saline control			Chronic ketamine (5 mg/kg)			Chronic ketamine (30 mg/kg)			Chronic MK-801 (0.02 mg/kg)			Chronic MK-801 (2 mg/kg)		
	Gamma power (nV^2^ Hz)	Peak power (nV^2^/Hz)	Peak freq. (Hz)	Gamma power (nV^2^ Hz)	Peak power (nV^2^/Hz)	Peak freq. (Hz)	Gamma power (nV^2^ Hz)	Peak power (nV^2^/Hz)	Peak freq. (Hz)	Gamma power (nV^2^ Hz)	Peak power (nV^2^/Hz)	Peak freq. (Hz)	Gamma power (nV^2^ Hz)	Peak power (nV^2^/Hz)	Peak freq. (Hz)
1 (0–5 s)	68.2 ± 24.7	10.4 ± 3.5	47.4 ± 1.2	59.2 ± 18.1	11.3 ± 4.4	41.5 ± 3.2	40.3 ± 8.7	6.5 ± 1.4	40.4 ± 1.3^b^	43.7 ± 13.1	6.6 ± 1.9	45.2 ± 1.1	57.2 ± 20.5	9.3 ± 3.2	49.6 ± 5.1
2 (5–10 s)	56.1 ± 14.2	13.1 ± 5.0	42.7 ± 1.7	46.6 ± 18.4	16.4 ± 6.0	37.6 ± 1.9	17.4 ± 4.3^b^	9.1 ± 1.5	35.3 ± 0.7^b^	39.1 ± 9.5	8.5 ± 2.2	40.5 ± 1.6	35.6 ± 8.9	7.6 ± 2.7	41.5 ± 0.7
3 (10–15 s)	30.8 ± 12.5	10.1 ± 4.6	37.0 ± 1.5	21.5 ± 9.5	20.5 ± 3.6	31.5 ± 3.1	8.5 ± 1.7^a^	8.7 ± 1.7	31.9 ± 0.4^b^	10.9 ± 2.3	8.6 ± 1.9	36.4 ± 1.5	9.8 ± 2.8	4.2 ± 1.3	36.2 ± 0.8
4 (15–20 s)	14.8 ± 5.1	6.7 ± 2.0	34.2 ± 1.3	13.9 ± 5.7	23.4 ± 7.3^a^	28.8 ± 3.4	5.0 ± 0.9^a^	6.1 ± 1.5	30.8 ± 0.3^b^	6.3 ± 2.2	4.0 ± 0.7	34.2 ± 1.4	2.8 ± 0.5	3.5 ± 1.7	33.8 ± 0.8
5 (20–25 s)	10.2 ± 3.8	5.9 ± 2.7	33.0 ± 0.9	8.3 ± 3.1	16.3 ± 5.9	25.1 ± 3.6^a^	3.6 ± 0.6	4.4 ± 1.2	26.0 ± 1.2^b^	3.5 ± 1.2	2.2 ± 0.5	32.6 ± 1.6	1.8 ± 0.3	1.2 ± 0.4	31.7 ± 1.4
6 (25–30 s)	4.9 ± 1.8	3.3 ± 1.9	33.0 ± 1.4	6.4 ± 2.5	11.2 ± 5.5	24.4 ± 3.7^a^	3.1 ± 0.5	3.7 ± 1.2	25.8 ± 1.2^b^	2.0 ± 0.5	1.6 ± 0.5	31.0 ± 1.5	1.2 ± 0.2	0.6 ± 0.2	29.3 ± 1.4

Data is the mean ± SEM.

^a^Value is significantly (*p* < 0.05) different from control.

^b^Value is significantly (*p* < 0.01) different from control.

Three measures from each PSD segment were used to characterize the oscillations: peak power, peak frequency, and total GBO power. Peak power was defined as the highest amplitude in the averaged PSD (>10 Hz, bins of 1.2 Hz). The frequency at which the peak power was observed was defined as the peak frequency. Total GBO power was determined by integration of averaged PSD between 40 and 50 Hz, which represents roughly the average peak frequency of the oscillatory response observed under control (no drug treatment) conditions ±5 Hz. This narrow frequency band was chosen to reduce variability of the PSD measures, when compared *between* groups of animals, i.e., drug treated vs. saline controls [note: this band is narrower than that used in our previous study (15) where we performed a within-animal (within-slice) comparison of the effects of NMDAR antagonists].

### Statistical analysis

Overall analysis of chronic drug effects were initially tested using repeated measures ANOVA. Further analyses of the results were performed using Student’s *t*-test for statistical comparison of individual epochs of oscillatory response. All statistical analysis was performed using SPSS 10 (SPSS Inc.). Differences were considered to be significant at *p* < 0.05. Averaged values reported in this manuscript are expressed as mean ± SEM.

## Results

### Chronic ketamine decreases the peak frequency of PrL GBO

To evaluate the effects of chronic ketamine on KA-induced oscillations, adult mice were given five daily, i.p. injections of ketamine, a treatment paradigm similar to that previously observed to elicit Sz-like alteration in neural circuitry (16, 23). Twenty-four hours after the final injection, KA-induced oscillations in layer II/III were characterized from PrL slices collected from either drug treated (*n* = 9) or control animals which received saline injections (*n* = 7). Visual inspection of a grand average of KA-elicited oscillations recorded from animals receiving chronic ketamine (30 mg/kg) injections revealed a clear reduction in the KA-elicited oscillatory response when compared to saline treated controls (Figure 1).

This data was initially analyzed using the method employed in our earlier acute NMDAR antagonist study (15), which examined the first 5 s of oscillatory activity immediately following the decay of the DC transient caused by KA application (2.5–7.5 s following KA application). This analysis revealed a significant (*t*_13_ = 3.74, *p* < 0.01) decrease in the peak frequency (saline: 47.4 ± 1.2 Hz; ketamine: 40.4 ± 1.3 Hz) of elicited oscillations in slices from chronic ketamine treated mice (Table 1: epoch 1). Both the peak power and total GBO power within the first 5 s also tended to decrease, but these effects were not statistically significant (*t*_13_ = 1.24, *p* = 0.24 and *t*_13_ = 1.18, *p* = 0.26 respectively).

Visual inspection of the grand averages suggested that oscillatory activity elicited in 30 mg/kg chronic ketamine treated slices decayed faster than in saline treated animals. Thus, to determine if chronic ketamine treatment impaired the ability of the PrL slice to maintain the KA-elicited GBO, we additionally analyzed the first 30 s of elicited oscillatory activity subdivided into 5 s epochs (Figure 2; Table 1). Using this analytical method, a repeated measures ANOVA observed a significant reduction in both the peak frequency of elicited oscillations (*F*_1,13_ = 52.77; *p* < 0.01), and total GBO power (*F*_1,13_ = 9.99; *p* < 0.01), across the full 30 s of KA-elicited activity. No effect was observed on peak power (*F*_1,13_ = 0.46; *p* = 0.51). Further statistical analysis, comparing each epoch individually, showed that the significant decrease in peak frequency observed above was maintained throughout all periods of analyzed activity. While total GBO power was reduced in chronic ketamine treated animals throughout the entire 30 s of analyzed activity, this reduction was only statistically significant in the second (5–10 s; *t*_13_ = 3.09, *p* < 0.01), third (10–15 s; *t*_13_ = 2.18, *p* < 0.05), and fourth (15–20 s; *t*_13_ = 2.30, *p* < 0.05) epochs. No significant change in peak power was seen during these periods.

Chronic treatment with a lower concentration of ketamine (5 mg/kg, *n* = 5), using the same dosing regimen described above conversely appeared to lead to a mild potentiation of the power of the KA-elicited oscillatory response (Figure 1). Nevertheless, analysis of elicited oscillations from these mice revealed that the significant decrease in peak frequency observed with 30 mg/kg ketamine was also evident at this lower dose (repeated measure ANOVA; *F*_1,9_ = 6.95; *p* < 0.05). This decrease was of a similar magnitude as observed with the higher ketamine dose when compared across all epochs of analyzed activity at 5 mg/kg, however, it was only significant in epochs 5 and 6 (*t*_9_ = 2.28, *p* < 0.05; *t*_9_ = 2.34, *p* < 0.05, respectively), and reached a trend level of significance in epochs 1, 2, 3, and 4 (*t*_9_ = 1.87, *p* = 0.10; *t*_9_ = 1.98, *p* = 0.08; *t*_9_ = 1.70, *p* = 0.13; *t*_9_ = 1.75, *p* = 0.12, respectively). 5 mg/kg ketamine did not lead to a decrease in the GBO power of KA-elicited oscillations (Figure 2). Similarly, no significant changes were found in peak power, compared to saline controls across the entire 30 s of elicited oscillations analyzed (repeated measures ANOVA). Comparison of individual epochs for total GBO and peak power did reveal a significant increase in peak power in the fourth epoch (15–20 s; *t*_9_ = −2.54; *p* < 0.05) and a trend level increase in the third (10–15 s; *t*_9_ = −1.73; *p* = 0.12) and fifth (20–25 s; *t*_9_ = −1.71; *p* = 0.12) epochs, however.

### Chronic MK-801 revealed no statistically significant effect on PrL GBO

While ketamine is commonly used recreationally and clinically to elicit Sz-like effects, several other drugs which inhibit NMDAR activity have also been used to elicit Sz-like behavioral effects in animals (8, 9). Thus, we also examined the effect of MK-801, a non-competitive NMDAR antagonist which is more selective than ketamine, since ketamine has effects on a number of other neurotransmitter receptors (15, 27, 28). Previous studies have shown that daily injections of 0.02 mg/kg MK-801, were sufficient to induce Sz-like alterations in neural circuitry, and behavioral effects (24). Thus, we gave mice five daily, i.p. injections of MK-801 (0.02 mg/kg) or saline, as above. Twenty-four hours after the final injection, KA-induced oscillations were characterized from PrL slices from both drug treated (*n* = 7) and saline control (*n* = 7) animals. As shown in Figure 1, the grand average of KA-elicited oscillations recorded in slices from MK-801 mice also suggested an overall reduction in elicited oscillatory response. Despite this, repeated measure ANOVA analysis of the elicited activity revealed no significant effect on total GBO power (*F*_1,11_ = 3.83; *p* = 0.08), peak frequency (*F*_1,11_ = 1.66; *p* = 0.22), or peak power (*F*_1,11_ = 0.97; *p* = 0.35). Comparing across individual epochs (Table 1), while the average GBO power was lower than that observed in saline controls (Figure 3A), these decreases reached only a trend level of significance in epochs 3, 4, 5, and 6 (*t*_11_ = 1.70, *p* = 0.12; *t*_11_ = 1.61, *p* = 0.14; *t*_11_ = 1.79, *p* = 0.10; *t*_11_ = 1.63, *p* = 0.13, respectively). Additionally, no significant (*p* > 0.05) effect was observed on peak power, or peak frequency across the entire range of analyzed KA-elicited activity (Figures 3B,C).

To ensure that the observed lack of effect was not caused by the dose of MK-801 being too low, the above experiments were repeated using first 0.5 mg/kg (*n* = 4; data not shown) and then 2 mg/kg (*n* = 3; Figure 3). As above, neither higher dose of MK-801 had a significant effect on total GBO power, peak power, or peak oscillatory frequency with either repeated measure ANOVA, or individual epoch analysis. Additionally, increasing the length of chronic drug treatment from 5 to 14 days of daily i.p. injections of 2 mg/kg MK-801 (*n* = 4) had no effect on *in vitro* PrL oscillatory activity compared to saline controls (data not shown).

## Discussion

The results reported in this study represent the first investigation of the effects of chronic (5 days) application of NMDAR antagonists, ketamine, and MK-801, on gamma band oscillations (GBO) in the adult mouse neocortex, *ex vivo*. There were three main findings: (1) Administration of chronic subanesthetic ketamine at 30 and 5 mg/kg reduced the peak frequency of the elicited oscillations; (2) Chronic ketamine at 30 mg/kg also reduced the power of cortical GBO within the 40–50 Hz band; (3) Somewhat surprisingly, the effect of ketamine was not mimicked by a more selective NMDAR antagonist, MK-801. In the following sections we discuss these three findings in more detail.

### Chronic ketamine reduced the peak oscillatory frequency of GBO

Several studies have suggested that the neocortical circuitry has an intrinsic resonance frequency (29, 30). Interestingly, each cortical area appears to have its own dominant frequency with frontal cortices showing a resonance frequency in the beta/gamma bands (30). Although most studies of GBO in Sz focus on changes in the power of oscillations, there is some evidence that Sz is also associated with a change (decrease) in the peak resonance frequency of cortical circuits. Several studies have reported a reduced 40 Hz response to auditory steady-state stimulation and a trend toward an increased response at 20 Hz (31, 32). Similarly, visual Gestalt stimuli elicit a lower frequency GBO response in schizophrenics than in healthy individuals (33). More recently, Tononi and colleagues used transcranial magnetic stimulation to probe the natural oscillatory frequency of cortical circuits in Sz (34). Interestingly, Sz subjects showed a significant slowing in the peak frequency of cortical oscillations, with the maximal decrease (10 Hz) occurring in the prefrontal cortex. Furthermore, the prefrontal natural frequency of individuals with Sz was slower than in any of the healthy control subjects and was correlated with both positive and negative symptoms.

While our previous findings with acute ketamine (15) are not directly comparable to our current chronic findings, due to the different routes of ketamine administration (bath application vs. systemic), it is striking that a similar decrease in the peak oscillatory frequency of KA-elicited oscillations was observed in both experiments. Thus, acute or chronic ketamine application reduced the natural resonance frequency of prefrontal cortical circuits, similar to findings in Sz patients (34). This downward shift in the oscillatory frequency would impair the ability of the circuit to receive and process input in the normal GBO range, as it could no longer reliably follow neuronal input at such high frequencies. This would result in reduced/inappropriate synchronization of neural activity, which would likely contribute to both the psychosis and impaired cognition observed in clinical studies, as well as Sz-like behaviors in animal studies.

### Chronic ketamine reduced the power of neocortical GBO

Many studies of GBO in Sz patients have shown reductions in the power of *evoked* GBO in a variety of sensory or cognitive paradigms (35, 36). Our results here in the mouse prefrontal cortex and those of Ferrarelli et al. (34), in Sz patients suggest that one reason for such decreased GBO power may be a reduction in the peak resonance frequency of cortical circuitry. Another reason may be increased spontaneous, *background broadband power* due to increased excitability of principal neurons, as observed in genetic models with reduced NMDAR expression in parvalbumin-positive interneurons [see Ref. (37–39)]. In our study, with 30 mg/kg chronic ketamine we did not observe a broadband increase in power, but rather a decrease in a narrow 40–50 Hz band around the peak resonance frequency, presumably due to the reduction in this peak frequency (see above). Similarly, *in vivo*, in adult animals, chronic ketamine reduced GBO activity in the hippocampus (13). In our study, with the lower dose of 5 mg/kg, the data plotted in Figures 1 and 2 appeared to show increased power compared to saline controls, particularly notable in the 15–20 s epoch. Despite this, statistical analysis (repeated measures ANOVA) indicated that overall this effect was not significant. Thus, we do not believe there is any physiological relevance to the elevated peak power measures observed with 5 mg/kg ketamine treatment. Together, these results with 30 and 5 mg/kg ketamine suggest that a higher dose is necessary to observe Sz-like reductions in GBO power.

### Chronic MK-801 application had no statistically significant effect on PrL GBO

Surprisingly, we found that chronic treatment with MK-801, a more specific NMDAR antagonist, did not reproduce the Sz-like impairment in elicited PrL GBO caused by chronic ketamine. Increasing the concentration of MK-801 or the duration of application did not change the response. Disparity in the efficacy of different NMDAR antagonists in mimicking Sz-like effects have also been observed in other studies. For instance, a recent study compared the behavioral effects of chronic PCP, a similarly promiscuous pharmacological agent to ketamine, and MK-801 in rodents (40). This study found that MK-801 treatment did not replicate the full spectrum of behavioral impairments induced by PCP. What accounts for these differences between Ketamine/PCP and MK-801? One possibility is that chronic suppression of NMDAR function by itself may not be enough to elicit the behavioral/electrophysiological effect. Ketamine, in particular, has actions on several other neurotransmitter receptors such as D_2_ receptors, 5-HT_2_ receptors, and GABA_A_ receptors (15, 27, 28, 41). Another possibility is that differences in the pharmacokinetics of these drugs affect the results. Thus, differences in the peak concentration, tissue penetration, and duration of action may affect the resultant changes in neocortical circuitry. While the precise mechanism has yet to be defined, our results, and those of others, suggest that chronic ketamine better models Sz-like changes in cortical function than chronic MK-801.

## Conclusion

Here we show, *ex vivo*, that chronic ketamine at 30 mg/kg results in Sz-like impairment of both the frequency and power of KA-elicited GBO in the PrL. This combined reduction in power and frequency is reminiscent of earlier clinical findings associated with cognitive deficits in Sz patients (31, 33–35). Our observation of these findings in an acute slice preparation provide strong evidence that the chronic ketamine mediated effects are mediated by local circuit alterations in the PrL. While important in establishing the locus of changes in oscillatory activity, one important limitation when comparing our *ex vivo* approach with clinical studies is that our slice preparation lacks input from other brain regions important in generating GBO activity *in vivo*. Thus, future studies testing the effect of chronic ketamine *in vivo*, utilizing transcranial magnetic stimulation, or other means, to generate GBO will be important for corroborating our results.

Acutely, NMDAR blockade leads to increased excitability of pyramidal neurons (42), altering the balance of excitation and inhibition in the cortical circuitry. Thus, we speculate that in our system chronic ketamine treatment leads to changes in the cortical circuitry in the PrL resulting over time in circuit dysfunction, perhaps through excitotoxicity (43) and/or upregulation of oxidative pathways (25). While we did not directly examine the cellular mechanisms behind this effect in this study, computational modeling suggests that either reductions in the number of PV interneurons or a reduction in GAD67 could account for our findings of reduced GBO power and peak frequency (44, 45). Our findings support the idea that deficits in executive function observed with chronic administration of ketamine in humans and animals (8, 9, 46–48) are due to an impaired ability of neocortical circuitry to generate and/or maintain the proper frequency oscillations and synchronicity necessary to bind together relevant information.

## Conflict of Interest Statement

The editor, Bernat Kocsis, and several authors of this manuscript (Ritchie Brown and Robert McCarley) recently coauthored a review article and have collaborated on funded and unfunded NIH research grants. However, Dr Kocsis was not involved in the design, execution, analysis or writing of the work presented in this manuscript.
